# Cystic Fibrosis Carrier States Are Associated With More Severe Cases of Bronchiectasis

**DOI:** 10.1093/ofid/ofae024

**Published:** 2024-01-17

**Authors:** Aaron C Miller, Logan M Harris, Kevin L Winthrop, Joseph E Cavanaugh, Mahmoud H Abou Alaiwa, Douglas B Hornick, David A Stoltz, Philip M Polgreen

**Affiliations:** Department of Internal Medicine, University of Iowa, Iowa City, Iowa, USA; Department of Biostatistics, University of Iowa, Iowa City, Iowa, USA; Department of Ophthalmology, Oregon Health and Science University, Portland, Oregon, USA; Department of Biostatistics, University of Iowa, Iowa City, Iowa, USA; Department of Internal Medicine, University of Iowa, Iowa City, Iowa, USA; Department of Internal Medicine, University of Iowa, Iowa City, Iowa, USA; Department of Internal Medicine, University of Iowa, Iowa City, Iowa, USA; Department of Internal Medicine, University of Iowa, Iowa City, Iowa, USA

**Keywords:** bronchiectasis, cystic fibrosis, carrier, heterozyote, phenotype

## Abstract

**Background:**

People with cystic fibrosis (CF) are at increased risk for bronchiectasis, and several reports suggest that CF carriers may also be at higher risk for developing bronchiectasis. The purpose of this study was to determine if CF carriers are at risk for more severe courses or complications of bronchiectasis.

**Methods:**

Using MarketScan data (2001–2021), we built a cohort consisting of 105 CF carriers with bronchiectasis and 300 083 controls with bronchiectasis but without a CF carrier diagnosis. We evaluated if CF carriers were more likely to be hospitalized for bronchiectasis. In addition, we examined if CF carriers were more likely to be infected with *Pseudomonas aeruginosa* or nontuberculous mycobacteria (NTM) or to have filled more antibiotic prescriptions. We considered regression models for incident and rate outcomes that controlled for age, sex, smoking status, and comorbidities.

**Results:**

The odds of hospitalization were almost 2.4 times higher (95% CI, 1.116–5.255) for CF carriers with bronchiectasis when compared with non–CF carriers with bronchiectasis. The estimated odds of being diagnosed with a *Pseudomonas* infection for CF carriers vs noncarriers was about 4.2 times higher (95% CI, 2.417–7.551) and 5.4 times higher (95% CI, 3.398–8.804) for being diagnosed with NTM. The rate of distinct antibiotic fill dates was estimated to be 2 times higher for carriers as compared with controls (95% CI, 1.735–2.333), and the rate ratio for the total number of days of antibiotics supplied was estimated as 2.8 (95% CI, 2.290–3.442).

**Conclusions:**

CF carriers with bronchiectasis required more hospitalizations and more frequent administration of antibiotics as compared with noncarriers. Given that CF carriers were also more likely to be diagnosed with *Pseudomonas* and NTM infections, CF carriers with bronchiectasis may have a phenotype more resembling CF-related bronchiectasis than non-CF bronchiectasis.

## Teaser

Using Marketscan data (2001-2021), we found that cystic fibrosis (CF) carriers with bronchiectasis face nearly 2.4 higher odds of hospitalization, increased rates of Pseudomonas and NTM infections and antibiotic use compared to non-CF-carriers with bronchiectasis suggesting a unique bronchiectasis phenotype.

Bronchiectasis, first described >200 years ago, is a chronic condition characterized by progressive lung damage, poor quality of life [1], and age-adjusted mortality that is twice that of the general population [2]. Most people with bronchiectasis experience a chronic cough, which is typically but not always productive [3]. Other symptoms include dyspnea, fatigue, wheezing, and pleuritic chest pain [4, 5]. Acute exacerbations of these chronic symptoms also occur, often associated with recurrent respiratory infections [3]. These chronic symptoms, along with the inflammation associated with exacerbations lead to the progressive and irreversible dilation of bronchi. As the disease progresses, the bronchiectatic airway interferes with effective mucociliary clearance, supporting bacterial colonization and leading to repeated cycles of infection and inflammation [6].

The prevalence of bronchiectasis in Europe and North America ranges between 163 and 566 per 100 000 people [2, 7], but limited access to diagnostic testing and a lack of epidemiologic data make it difficult to estimate the prevalence worldwide [8]. More recent reports indicate that the condition may be increasing in incidence [2, 9]. It typically occurs among people with an underlying host defect, which puts them at risk for recurrent respiratory infections [10, 11]. In North America and Europe, cystic fibrosis (CF) is a common and severe cause of bronchiectasis. Among the less common causes of bronchiectasis are defects in the systemic immune system (eg, common variable immune deficiency), deficiencies in mucociliary clearance (eg, primary ciliary dyskinesia), and other genetic diseases (eg, alpha-1 antitrypsin deficiency).

Bronchiectasis is so highly associated with CF that bronchiectasis in people without CF has been referred to as non-CF bronchiectasis [12]. People with CF experience chronic and recurrent respiratory infections and, as a result, commonly develop bronchiectasis starting at an early age [13, 14]. Because CF is an autosomal recessive disease, people with CF have 2 mutations in the *CFTR* gene (CF transmembrane conductance regulator). This gene encodes a chloride and bicarbonate channel [15–17], and people with 1 *CFTR* mutation (CF carriers) were traditionally thought not to be at risk for diseases associated with CF [18, 19]. However, in recent years, multiple reports indicate that CF carriers are at increased risk for multiple CF-associated diseases, including bronchiectasis [20], and multiple investigators have suggested that the CF carrier state may increase the risk for bronchiectasis [21–23]. If CF carriers are indeed at increased risk for complications of bronchiectasis, they may exhibit a phenotype more similar to CF than to other causes of bronchiectasis.

The purpose of this study is to compare health care outcomes between cases of bronchiectasis among CF carriers and cases of bronchiectasis among those who are not CF carriers. Specifically, our goal is to determine if CF carriers with bronchiectasis are more likely to be hospitalized, use more antimicrobials, and be diagnosed with infections from pathogens commonly associated with CF—specifically, *Pseudomonas aeruginosa* and nontuberculous mycobacteria (NTM).

## METHODS

### Data Source

We used data from the IBM MarketScan Research Databases from 2001 through 2021. We included data from the Commercial Claims and Encounters and Medicare Supplemental databases. These databases contain longitudinal insurance claims data from >195 million enrollees across the United States with employer-sponsored commercial health insurance or supplemental Medicare plans. Claims are provided for inpatient, outpatient, and emergency department encounters, and outpatient prescription medications.

### Study Population and Design

To compare outcomes between a cohort of CF carriers and a cohort of noncarriers following a diagnosis of bronchiectasis, we started by identifying all enrollees who had a bronchiectasis diagnosis based on the *ICD-9* code 494.X and *ICD-10* code J47.X. Next, we identified all enrollees diagnosed as a CF carrier through genetic screening using the diagnosis codes V8381 (*ICD-9-CM*) and Z141 (*ICD-10-CM*). We then identified noncarrier controls without diagnoses of *CFTR* mutations (ie, not diagnosed with CF or as a CF carrier). To minimize the inclusion of enrollees with undetected *CFTR* mutations, we also excluded from the control cohort any people in a family where another family member was diagnosed as a CF carrier or a patient with CF. Family members of patients with CF were not included among controls to minimize misclassification. Because many CF carriers are not identified, it is likely that any person who is not tested and therefore classified as a non–CF carrier in a family with patients with CF has a high likelihood of being a carrier. For hospitalization outcomes, only individuals with a primary bronchiectasis diagnosis were considered. *P aeruginosa* infections were identified with codes 0417 and 4821 (*ICD-9-CM*) and B965 and J151 (*ICD-10-CM*), and for NTM infections, we used 310 and 319 (*ICD-9-CM*), and A310 and A319 (*ICD-10-CM*).

### Statistical Analysis

We compared the incidence of *P aeruginosa* and NTM infections as well as the rate of occurrence of hospitalizations between CF carriers and noncarriers following a bronchiectasis diagnosis. We also compared the rate of antibiotic usage in the population with respect to the total number of unique visits where antibiotics were prescribed, the total number of unique antibiotics based on National Drug Codes and visit combinations, and the total number of days supplied.

Two primary models were used to analyze hospitalizations, *P aeruginosa* infections, NTM infections, and antibiotic usage, depending on the type of outcome. Both approaches used generalized linear models. The first model was a logistic regression model based on a binomial family with a logit link, and it was used to model incidence as a binary outcome. Specifically, logistic models were used to estimate the impact of CF carrier status on the odds of any occurrence of the outcome under evaluation as well as the incidence of hospitalization and *P aeruginosa* and NTM infections. The second model used a quasi-Poisson distribution with a log link for numeric outcomes. This model was used to estimate the rate ratio for the count of occurrences associated with CF carrier state and the total number of hospitalizations per individual because these outcomes tend to be overdispersed. For antibiotic usage, the total number of unique visits where antibiotics were prescribed, the total number of distinct antibiotics prescribed based on National Drug Codes, and the total days supplied of antibiotics were also modeled with the quasi-Poisson model.

In addition to evaluating the effect of the CF carrier state on the various outcomes, each model controlled for several other patient factors that may be associated with risk for more severe cases of bronchiectasis. Indicators were included per the Elixhauser comorbidity indicators (Agency for Healthcare Research and Quality), which were constructed with all inpatient and outpatient visits in the year prior to the index case of bronchiectasis. Additionally, models were adjusted for age, sex, smoking status, and the amount of enrollment time following the index bronchiectasis diagnosis. Smoking status was identified by all inpatient and outpatient visits in the year prior according to codes developed by the University of Wisconsin Center for Tobacco Research and Intervention [24].

### Sensitivity Analysis

We investigated if the identification of CF carriers through genetic testing could induce a type of confounding and/or misclassification bias—specifically, if patients more likely to receive genetic testing (eg, identified CF carriers) were more likely to receive other types of disease screening/testing (eg, cultures for NTM or *Pseudomonas*). In addition, patients with severe bronchiectasis might be more likely to be tested for CF. To investigate these possibilities, we repeated our analysis using a subset of control patients who received genetic testing for suspicion of *CFTR* mutations but were determined not to be CF carriers.

## RESULTS

A summary of the study cohort is given in Table 1. We identified 300 188 cases of bronchiectasis among patients without CF, which included 105 CF carriers and 300 083 controls who ranged in age from 0 to 106 years. Carriers tended to be younger and were more likely to be female as compared with controls, reflecting the fact that pregnant women are more likely to receive CF carrier testing. Table 1 also provides baseline counts of the primary study outcomes: when compared with controls, carriers were more likely to be hospitalized, have *P aeruginosa* or NTM infections, and receive more outpatient antibiotics.

**Table 1.** Study Population: Demographics, Hospitalization, Infection, and Antibiotic Summaries

	Controls With Bronchiectasis (n = 300 083)	CF Carriers With Bronchiectasis (n = 105)	Overall (N = 300 188)
Sex			
Male	118 763 (39.6)	29 (27.6)	118 792 (39.57)
Female	181 320 (60.4)	76 (72.4)	181 396 (60.43)
Age			
Mean (SD)	61.6 (19.3)	48.4 (17.4)	61.5 (19.3)
Median [range]	63.0 [0–106.0]	51.0 [0–82.0]	63.0 [0–106.0]
Enrollment time after bronchiectasis diagnosis, mo			
Mean (SD)	30.9 (31.6)	43.0 (37.8)	30.9 (31.7)
Median [range]	21.0 [1.0–242.0]	32.0 [1.0–157.0]	21.0 [1.0–242.0]
No. of primary bronchiectasis hospitalizations			
Mean (SD)	0.036 (0.291)	0.086 (0.370)	0.036 (0.291)
Median [range]	0 [0–24]	0 [0–3]	0 [0–24]
None (0)	291 853 (97.3)	98 (93.3)	291 951 (97.3)
1	6895 (0.024)	6 (0.057)	6901 (0.0230)
2	839 (0.003)	0 (0.000)	839 (0.003)
3	239 (0.001)	1 (0.010)	240 (0.001)
≥ 4	257 (0.001)	0 (0.000)	257 (0.001)
Patients with infection			
* Pseudomonas aeruginosa*	10 808 (3.6)	14 (13.3)	10 822 (3.6)
NTM	14 623 (4.9)	23 (21.9)	14 646 (4.9)
Antibiotics			
Distinct fill dates			
Mean (SD)	5.9 (11.5)	16.4 (24.5)	5.9 (11.5)
Median [range]	2 [0–660]	7 [0–141]	2 [0–660]
Distinct NDCs			
Mean (SD)	6.2 (12.3)	18.1 (29.0)	6.2 (12.3)
Median [range]	2 [0–660]	7 [0–188]	2 [0–660]
Days supplied			
Mean (SD)	86.5 (262.5)	337.8 (710.6)	86.6 (262.8)
Median [range]	14.0 [0–11369]	54.0 [0–4948]	14.0 [0–11369]

Data are presented as No. (%) unless noted otherwise.

Abbreviations: CF, cystic fibrosis; NCD, National Drug Code; NTM, nontuberculous mycobacteria.

Table 2 provides the regression results for hospitalization outcomes. In the logistic regression analysis, the odds of a primary bronchiectasis hospitalization were 2.42 times higher (95% CI, 1.12–5.26) for CF carriers vs controls after controlling for age, sex, smoking status, and comorbidities. When we considered the total number of hospitalizations per individual using the quasi-Poisson equivalent, the rate ratio was estimated to be 1.66 (95% CI, .53–5.29). Supplementary Tables 1 and 2 report counts and regression results, respectively, for all bronchiectasis-related hospitalizations and any hospitalization; these results also suggest that CF carriers were more likely to have any bronchiectasis hospitalization.

**Table 2.** Hospitalization Results: Generalized Linear Model With a Quasi-Poisson Distribution for Counts and Logistic Distribution for Binary Outcomes

	Hospitalization	
Coefficient	Yes/No, Odds Ratio (95% CI)	Count, Relative Risk (95% CI)
Carrier	2.421 (1.116–5.255)	1.658 (.530–5.285)
Age	1.003 (1.002–1.005)	0.996 (.994–.998)
Female sex	1.255 (1.197–1.316)	1.214 (1.132–1.303)
Smoking status	1.078 (1.009–1.151)	1.139 (1.031–1.259)
Time, mo	1.008 (1.008–1.009)	…^a^

^a^Model includes an offset term for the total number of enrollment months.

Table 3 provides regression results for the infection outcomes. The estimated odds of being diagnosed with a *P aeruginosa* infection for CF carriers vs noncarriers was about 4.27 times higher (95% CI, 2.42–7.55). The corresponding model for NTM estimated an odds ratio of 5.47 (95% CI, 3.40–8.80) for CF carriers as compared with controls without the CFTR mutation.

**Table 3.** Infection Results for *Pseudomonas* and Nontuberculous Mycobacterial Infections: Generalized Linear Model With a Logistic Distribution

	Odds Ratio (95% CI)	
Coefficient	*Pseudomonas*	Nontuberculous Mycobacterial Infection
Carrier	4.272 (2.417–7.551)	5.470 (3.398–8.804)
Age	1.004 (1.002–1.005)	1.017 (1.016–1.018)
Sex	0.952 (.915–.992)	2.018 (1.94–2.1)
Smoking status	1.107 (1.046–1.171)	0.908 (.86–.959)
Time	1.008 (1.008–1.009)	1.007 (1.007–1.008)

Supplementary Table 3 provides a more granular breakdown of the distribution in the number episodes of NTM and *Pseudomonas* infections between cases and controls; consistent with our primary model, CF carriers had a greater frequency of these infection episodes.

Table 4 provides regression results for antibiotic usage. In all 3 antibiotic models, the effect of carrier status was highly significant after controlling for age, sex, smoking status, enrollment time, and comorbidities. When compared with controls, carriers demonstrated a rate of distinct fill dates that was 2.01 (95% CI, 1.74–2.33) times higher; their distinct National Drug Codes were 2.13 (95% CI, 1.84–2.47) times higher; and their total number of days supplied was 2.80 (95% CI, 2.29–3.44) times higher.

**Table 4.** Antibiotic Results: Generalized Linear Model With a Quasi-Poisson Distribution

	Antibiotics, Relative Risk (95% CI)		
Coefficient	Distinct Fill Dates	Distinct NDCs	Days Supplied
Carrier	2.012 (1.735–2.333)	2.130 (1.835–2.473)	2.801 (2.290–3.442)
Age	0.999 (.999–1.000)	0.999 (.999–.999)	0.999 (.998–.999)
Sex	1.093 (1.082–1.103)	1.084 (1.073–1.095)	1.007 (.991–1.024)
Smoking status	1.015 (1.000–1.031)	1.015 (.999–1.031)	0.904 (.881–.928)

Estimated relative risks of number of filled antibiotic prescriptions, number of distinct antibiotics, and number of days of antibiotics filled.

Abbreviation: NDC, National Drug Code.

We performed a sensitivity analysis to determine if our findings could be explained by a confounding effect of whether the identified CF carriers were more likely to be screened for infectious diseases. Supplementary Table 4 presents the results where we repeated each of our analyses using a subset of 3893 controls who also received genetic testing. Across nearly all outcomes, we found consistent results that CF carriers were more likely to have *Pseudomonas* or NTM infections and greater antibiotic usage.

## DISCUSSION

We found that among people with bronchiectasis, CF carriers, as opposed to non–CF carriers, were more likely to be hospitalized with a primary diagnosis of bronchiectasis, prescribed a greater number of antimicrobial agents, and diagnosed with *P aeruginosa* and NTM infections. Collectively, our results suggest that CF carriers with bronchiectasis exhibit different clinical features than non–CF carriers and may share some of the features typical of CF-associated bronchiectasis. These findings may have important implications for personalizing treatment approaches for cases of bronchiectasis among CF carriers.

The potential health risks attributable to the CF carrier state have been debated for decades [25]. Early studies in the 1960s did not find that CF carriers had elevated risk for CF-related conditions, but these studies were relatively small [25]. More recent and larger case-control studies indicate that CF carriers are at increased risk for respiratory infections [26] and a range of other CF-related conditions [20]. Furthermore, CF-related health conditions with the highest incidence among people with CF, including bronchiectasis, are common among CF carriers [20]. Finally, prior studies of CF carriers, focused on bronchiectasis, showed that CF carriers are highly prevalent among people with bronchiectasis as compared with the general population [27–29].

Unlike most prior studies considering CF carriers and bronchiectasis, our study design included a control group of cases not identified as CF carriers, allowing us to describe phenotypes for CF carriers distinct from non–CF carriers. Two differences that we identified—more hospitalizations for bronchiectasis and a greater risk of infections by specific organisms—are consistent with a previously described “more frequent exacerbator” phenotype [30, 31].

Another previously described bronchiectasis phenotype involves infection or colonization with *P aeruginosa* [7], and we found that CF carriers were more likely to be diagnosed with a *Pseudomonas* infection. Interestingly, *P aeruginosa* in the context of bronchiectasis is associated with a higher degree of morbidity and mortality and a more accelerated progression of disease [32–34]. This finding is consistent with our finding that CF carriers were more likely to be hospitalized and receive antibiotics. Our finding that CF carriers are more likely to be diagnosed with a *P aeruginosa* infection is not surprising considering that infections with *P aeruginosa* are relatively common among people with CF [35]. NTM infections are also relatively common among people with CF [35].

People with CF have multiple reasons for developing bronchiectasis, including defects in mucociliary clearance [36–38] as well as acquired defects in their innate immune system (ie, reduced ability of defenses in the airway surface liquid to kill bacteria) [39, 40]. In addition, relative neutrophil dysfunction in the airway of people with CF may contribute to an excessive immune response leading to chronic inflammation and resulting airway damage [41]. Some or all of these phenomena could be at play in CF carriers albeit to a much lesser extent. Further study will be needed to understand why CF carriers are at increased risk for developing bronchiectasis. Because a minority of CF carriers ultimately develop bronchiectasis, the pathogenesis likely relies on additional genetic and environmental factors. Perhaps the reduced level of CFTR function acts in an additive or synergistic fashion with environmental or genetic factors. Accordingly, studying specific risk factors and comparing CF carriers who develop bronchiectasis with CF carriers who do not may accelerate the discovery of other genetic and/or environmental risk factors for bronchiectasis.

Our results may have 2 implications for future treatment approaches. First, treatment approaches developed to treat patients with CF-associated bronchiectasis have not typically been successful in trials to treat non–CF-related bronchiectasis. For example, DNase (dornase alpha)—a mucolytic developed for patients with CF that decreased the frequency of exacerbations and the loss of FEV1—resulted in an increased risk of exacerbations when tested in patients with non-CF bronchiectasis [42]. In addition, inhaled mannitol improved FEV1 among people with CF [43, 44] but not among people with non-CF bronchiectasis [43]. Second, highly effective modulators are available for CF that restore CFTR function. Elexacaftor-tezacaftor-ivacaftor, widely used since the Food and Drug Administration’s release in October 2019, increases CFTR function for those with at least 1 F508del *CFTR* mutation, which is 86% of the US population with CF [45]. Data from clinical trials and real-world evidence [35] show that elexacaftor-tezacaftor-ivacaftor dramatically decreases infection-related episodes among people with CF. While this treatment is incredibly expensive, it is conceivable that it could play a role in treating specific CF carriers, such as those with bronchiectasis chronically infected by *P aeruginosa* or NTM, and should be explored in future work.

Most of the limitations of our work are related to the use of administrative data, which are subject to the sensitivity and specificity of specific *ICD-9-CM* and *ICD-10-CM* codes. Thus, we do not have access to specific microbiology tests, clinical notes, radiology reports, or spirometry reports. As a result, we were unable to use some outcome measures commonly used to study bronchiectasis. In addition, we cannot determine the severity of bronchiectasis at baseline. Also, we do not know the specific *CFTR* genotype in our population, but it should reflect the distribution of the CF population, where the majority has at least 1 F508del *CFTR* mutation [46]. Another limitation of our work relates to the age and sex distribution of our cohort. Many CF carriers in our population were identified through prenatal genetic counseling or newborn screening efforts. Accordingly, the CF carriers in our cohort were relatively young and disproportionally female. If our study included older patients, we might expect to discover a higher rate of hospitalization among CF carriers.

Another limitation of our observational design is that we identified only a minority of CF carriers. A substantial proportion of CF carriers are misclassified as non–CF carriers, and the carriers that we did identify may differ in meaningful ways from those who are misclassified. For example, it is possible that our carriers were universally more likely to receive disease screening, including genetic testing for CF and testing for infectious diseases. While random misclassification of CF carriers among controls may bias our results toward the null hypothesis, a type of confounding by indication, where identified CF carriers have a greater propensity to receive disease testing, may bias results away from the null hypothesis. We conducted a sensitivity analysis using only 3893 controls who received genetic screening for “suspected CFTR mutations” and found that nearly all of the primary outcomes remained significantly greater among carriers relative to controls. While these effect estimates decreased slightly from the primary analysis, this is likely explained by such control patients having more severe disease on average: most of the identified CF carriers were tested in the course of pregnancy, whereas controls tested for “suspected CFTR mutations” are generally tested in response to recurrent CF-related conditions. Thus, while it is possible that our primary effect estimates might be slightly biased high or low, we did not find evidence that they were entirely driven by misclassification of controls.

Despite our limitations, we show that CF carriers are at risk for complications of bronchiectasis as compared with non–CF carriers and that they are also at increased risk for pathogens associated with more severe or aggressive cases of bronchiectasis. Given that there are at least 10 million CF carriers in the United States alone and that a substantial proportion of people with bronchiectasis have CFTR mutations [27, 28, 47, 48], a better understanding is needed of the role that CFTR function may play in the development of bronchiectasis. Such an understanding may help personalize therapies for specific endotypes of bronchiectasis for CF carriers and non–CF carriers. Indeed, CF carrier testing should be included in bronchiectasis workups. Finally, because most CF carriers do not develop bronchiectasis, there is a need to identify other environmental factors and genetic mutations that may increase the risk for bronchiectasis among CF carriers.

## Supplementary Material

ofae024_Supplementary_Data

## Supplementary Data

Supplementary materials are available at *Open Forum Infectious Diseases* online. Consisting of data provided by the authors to benefit the reader, the posted materials are not copyedited and are the sole responsibility of the authors, so questions or comments should be addressed to the corresponding author.

## Notes

***Patient consent statement*.** This study has been deemed non–human subjects research by the University of Iowa Institutional Review Board.

***Financial support.*** This work was supported by the National Institute of Allergy and Infectious Diseases (grant R01AI143671) ; and National Center for Advancing Translational Sciences (grant UL1TR002537).
