# Supplementary material for: Cystic Fibrosis Carrier States Are Associated With More Severe Cases of Bronchiectasis
Source: Open Forum Infect Dis. 2024 Jan 17;11(2):ofae024. doi: 10.1093/ofid/ofae024 (PMC10883289; doi:10.1093/ofid/ofae024)
Supplement: ofae024_Supplementary_Data [file ofae024_supplementary_data.docx]

**Supplementary Table 1** – Hospitalization counts with or without a bronchiectasis diagnosis

| **Number of Visits** | **Cases** | **Controls** | **Combined** |
| --- | --- | --- | --- |
| **Primary Bronchiectasis Hospitalization** | | | |
| **Mean (SD)** | 0.086 (0.370) | 0.036 (.291) | .036 (.291) |
| **Median [Min, Max]** | 0 [0, 3] | 0 [0, 24] | 0 [0, 24] |
| **No Visits** | 98 (93.3%) | 291,853 (97.3%) | 291,951 (97.3%) |
| **1 Visit** | 6 (0.057%) | 6,895 (0.024%) | 6,901 (0.0230%) |
| **2 Visits** | 0 (0.000%) | 839 (0.003%) | 839 (0.003%) |
| **3 Visits** | 1 (0.010%) | 239 (0.001%) | 240 (0.001%) |
| **≥4 Visits** | 0 (0.000%) | 257 (0.001%) | 257 (0.001%) |
| **Any Bronchiectasis Hospitalization** | | | |
| **Mean (SD)** | 0.438 (1.33) | 0.225 (0.697) | 0.225 (0.697) |
| **Median [Min, Max]** | 0 [0,12] | 0 [0,41] | 0 [0,41] |
| **No Visits** | 80 (0.762) | 250360 (0.834) | 250440 (0.834) |
| **1 Visit** | 17 (0.162) | 40881 (0.136) | 40898 (0.136) |
| **2 Visits** | 4 (0.038) | 5292 (0.018) | 5296 (0.018) |
| **3 Visits** | 3 (0.029) | 1696 (0.006) | 1699 (0.006) |
| **≥4 Visits** | 1 (0.01) | 1854 (0.006) | 1855 (0.006) |
| **Any Hospitalization** | | | |
| **Mean (SD)** | 2.21 (4.51) | 2.03 (3.31) | 2.03 (3.31) |
| **Median [Min, Max]** | 1 [0,29] | 1 [0,122] | 1 [0,122] |
| **No Visits** | 44 (0.419) | 118576 (0.395) | 118620 (0.395) |
| **1 Visit** | 26 (0.248) | 63437 (0.211) | 63463 (0.211) |
| **2 Visits** | 13 (0.124) | 37773 (0.126) | 37786 (0.126) |
| **3 Visits** | 7 (0.067) | 24172 (0.081) | 24179 (0.081) |
| **≥4 Visits** | 15 (0.143) | 56125 (0.187) | 56140 (0.187) |

**Supplementary Table 2** – Regression for hospitalization with or without a bronchiectasis diagnosis

| **Coefficient** | **Hospitalization (Yes/No)** | **Hospitalization (Count)** |
| --- | --- | --- |
| **Primary Bronchiectasis Hospitalization** | | |
| **Carrier** | 2.421 (1.116,5.255) | 1.658 (0.530,5.285) |
| **Age** | 1.003 (1.002,1.005) | 0.996 (0.994,0.998) |
| **Female Sex** | 1.255 (1.197,1.316) | 1.214 (1.132,1.303) |
| **Smoking Status** | 1.078 (1.009,1.151) | 1.139 (1.031,1.259) |
| **Time (Months)** | 1.008 (1.008,1.009) | N/A* |
| **Any Bronchiectasis Hospitalization** | | |
| **Carrier** | 1.984 (1.252,3.144) | 1.662 (0.944,2.928) |
| **Age** | 1.006 (1.005,1.006) | 1.003 (1.002,1.003) |
| **Female Sex** | 1.017 (0.996,1.039) | 0.995 (0.965,1.027) |
| **Smoking Status** | 1.453 (1.415,1.493) | 1.427 (1.371,1.485) |
| **Time (Months)** | 1.005 (1.004,1.005) | N/A* |
| **Hospitalization for any reason** | | |
| **Carrier** | 1.293 (0.861,1.942) | 1.108 (0.692,1.775) |
| **Age** | 1.019 (1.019,1.020) | 1.010 (1.010,1.011) |
| **Female Sex** | 0.808 (0.795,0.822) | 0.837 (0.822,0.853) |
| **Smoking Status** | 1.895 (1.850,1.941) | 1.531 (1.495,1.568) |
| **Time (Months)** | 1.013 (1.012,1.013) | N/A* |

**Supplementary Table 3** – Distribution of visits and episodes for NTM and *Pseudomonas* infections between cases and controls. Distinct episodes were defined by events separated by at least 90 or 180 days between visits with a given diagnosis (i.e., all visits with an NTM diagnosis within 90 or 180 days of another were treated as the same episode)

|  | **Cases** | **Controls** |
| --- | --- | --- |
| Pseudomonas Infections | | |
| **Number of Enrollees (%)** | 14 (13.3%) | 10,808 (3.60%) |
| **Number of Distinct Visit Dates** | 121 | 59,614 |
| **Total Number of Episodes (separated by 90 days)** | 21 | 15,913 |
| **Number of patients w/given number of episodes (separated by 90 days)** |  |  |
| **0 Episodes** | 91 (86.67%) | 289275 (96.4%) |
| **1 Episode** | 9 (8.57%) | 7929 (2.64%) |
| **2 Episodes** | 4 (3.81%) | 1697 (0.57%) |
| **3 Episodes** | 0 (0.00%) | 646 (0.22%) |
| **4 Episodes** | 1 (0.95%) | 289 (0.1%) |
| **≥5 Episodes** | 0 (0.00%) | 247 (0.08%) |
| **Total Number of Episodes (separated by 180 days)** | 21 | 13,808 |
| **Number of patients w/given number of episodes (separated by 180 days)** |  |  |
| **0 Episodes** | 91 (86.67%) | 289275 (96.4%) |
| **1 Episode** | 9 (8.57%) | 8688 (2.9%) |
| **2 Episodes** | 4 (3.81%) | 1507 (0.5%) |
| **3 Episodes** | 0 (0.00%) | 430 (0.14%) |
| **4 Episodes** | 1 (0.95%) | 129 (0.04%) |
| **≥5 Episodes** | 0 (0.00%) | 54 (0.02%) |
| NTM Infections | | |
| **Number of Enrollees (%)** | 23 (21.9%) | 14,623 (4.87%) |
| **Number of distinct Visit Dates** | 803 | 135,627 |
| **Total Number of Episodes (separated by 90 days)** | 86 | 36,475 |
| **Number of patients w/given number of episodes (separated by 90 days)** |  |  |
| **0 Episodes** | 82 (78.1%) | 285460 (95.13%) |
| **1 Episode** | 6 (5.71%) | 6949 (2.32%) |
| **2 Episodes** | 5 (4.76%) | 2927 (0.98%) |
| **3 Episodes** | 4 (3.81%) | 1672 (0.56%) |
| **4 Episodes** | 2 (1.9%) | 1075 (0.36%) |
| **≥5 Episodes** | 6 (5.71%) | 2000 (0.67%) |
| **Total Number of Episodes (separated by 180 days)** | 37 | 24,276 |
| **Number of patients w/given number of episodes (separated by 180 days)** |  |  |
| **0 Episodes** | 82 (78.1%) | 285460 (95.13%) |
| **1 Episode** | 13 (12.38%) | 9475 (3.16%) |
| **2 Episodes** | 8 (7.62%) | 2858 (0.95%) |
| **3 Episodes** | 1 (0.95%) | 1192 (0.4%) |
| **4 Episodes** | 0 (0.00%) | 552 (0.18%) |
| **≥5 Episodes** | 1 (0.95%) | 546 (0.18%) |

**Supplementary Table 4** – Sensitivity results of regression analyses when repeated using a subset of 3,893 control patients who also received genetic testing for suspected CFTR mutations. Control patients who received genetic testing were identified using the CPT codes 81220, 81479, 81221, 81222, 81223, 81224, 81412. Note: these estimates decreased slightly from the primary analysis; however, control patients who received one of these genetic screening codes may have more severe disease state compared to identified case patients. Most CF carriers in our analysis were identified in the course of pregnancy/procreative care, whereas these CPT codes are generally used when a patient is suspected of having CF (e.g., recurrent respiratory infections).

| **Outcome** | **Regression Estimate** |
| --- | --- |
| **Pseudomonas** | 2.554 (1.408, 4.633) |
| **Non-TB Mycobacterial Infection** | 2.224 (1.350, 3.664) |
| **Primary Bronchiectasis Hospitalization (Any Visit)** | 1.627 (0.722, 3.668) |
| **Primary Bronchiectasis Hospitalization (Count of Visits)** | 1.027 (0.329, 3.209) |
| **Antibiotics (Distinct Fill Dates)** | 1.456 (1.227, 1.728) |
| **Antibiotics (Distinct NDC Codes)** | 1.541 (1.291, 1.838) |
| **Antibiotics (Days Supplied)** | 1.824 (1.442, 2.308) |
